# The Effect of Sn Addition on Zn-Al-Mg Alloy; Part II: Corrosion Behaviour

**DOI:** 10.3390/ma14185290

**Published:** 2021-09-14

**Authors:** Zuzana Gabalcová, Peter Gogola, Martin Kusý, Henrich Suchánek

**Affiliations:** Faculty of Materials Science and Technology in Trnava, Institute of Materials Science, Slovak University of Technology in Bratislava, Ulica Jána Bottu 25, 917 24 Trnava, Slovakia; peter.gogola@stuba.sk (P.G.); martin.kusy@stuba.sk (M.K.); henrich.suchanek@stuba.sk (H.S.)

**Keywords:** Zn-based alloy, Sn-addition, corrosion products, salt spray test, intergranular corrosion, corrosion penetration depth, weight loss

## Abstract

Corrosion behaviour of Sn (0.0, 0.5, 1.0, 2.0 and 3.0 wt.%)-doped Zn 1.6 wt.% Al 1.6 wt.% Mg alloys exposed to salt spray testing was investigated. Intergranular corrosion was observed for all alloys in both as-cast and annealed states. However, due to microstructure spheroidisation in the annealed samples, potential intergranular corrosion paths are significantly reduced. Samples with 0.5 wt.% of Sn showed the best corrosion properties. The main corrosion products identified by XRD analysis for all samples were simonkolleite and hydrozincite. Occasionally, ZnO and AlO were identified in limited amounts.

## 1. Introduction

A wide range of commercial Zn-based hot-dip coatings are used for corrosion protection. These also include Zn-Al-Mg-based coatings such as Magizinc (MZ) with Zn 1.6 wt.% Al and 1.6 wt.% Mg. It is widely used in the coating industry including steel sheet production for building, energetics, and the automotive industry [1,2,3,4,5,6,7,8,9,10,11,12].

Neutral salt spray testing (NSST) is used as an industry standard for corrosion resistance testing. Zn-Al-Mg coatings perform notably better compared to conventional hot-dip Zn coatings. The presence of Mg in the Zn-Al-Mg coatings enables the stabilisation of protective corrosion products like simonkolleite and hydrozincite [13,14,15,16]. Regarding the microstructure, Mg addition to binary Zn-Al alloys results in the formation of intermetallic phases such as Zn_2_Mg and Zn_11_Mg_2_. These phases are more corrosion active even compared to the η(Zn) phase, hence enabling the more effective cathodic protection of steel substrates [17]. They are formed within eutectics in the interdendritic areas of primary η(Zn) dendrites. Unfortunately, these phases are also enabling the cathodic protection of this Zn-based matrix, hence overall corrosion attack starts as the intergranular (IG) corrosion. Sources have reported this phenomenon, however only on the coatings with a limited thickness of up to 50 µm. In all these corrosion test results, substantial parts of the coatings were affected by IG corrosion locally, even across the entire coating [18,19,20].

The potentials of additional alloying of Zn-Al-Mg systems by Cr, Zr, Ti Mo, Mn, Si, etc. have been already studied in the literature [10]. Sn is also an interesting candidate due to its high affinity to Mg [21]. The preliminary research [12] into the development of microstructure and corrosion resistance of the Zn-Al-Mg + Sn alloy system has shown that Sn can affect the phase composition, and consequently the corrosion properties of MZ. In the follow-up to these results, this system is being investigated with an extended experimental scope in Parts I and II of the current articles. The main aim of these additional experiments is to observe if long time exposure to rather high temperatures (1 h at 310 °C) have a significant influence on the corrosion properties of these alloys. Based on these inputs, bulk samples were chosen for our research. This enabled to investigate the IG corrosion phenomena for these alloys in both as-cast and annealed states without the limit of a coating’s thickness.

## 2. Materials and Methods

As already described in Part I of this article [22], five different alloys with the designed nominal composition of Zn-1.6Al-1.6Mg-xSn (wt.%), where x = 0.0, 0.5, 1.0, 2.0 and 3.0 wt.%, respectively, were prepared by melting pure Zn at 470 °C and mixing in the appropriate amount of a 50 wt.% Al + 50 wt.% Mg master alloy. These raw materials were preheated to 400 °C to facilitate their rapid melting. Due to the low melting point of Sn, it was added in the last step. Table 1 indicates that the measured chemical compositions of the alloys by glow discharge optical emission spectroscopy (GDOES, Spectruma GDA 750, Spectruma Analytik GmbH, Hof, Germany) are in a good agreement with the nominal ones.

As a reference material for the corrosion test, 4N5 purity Zn-samples were cast. Two types of cylindrical samples were prepared for each alloy: (i) as-cast samples and (ii) cast and subsequently solution annealed at 310 °C for 1 h.

Casting was done from 470 °C of melt temperature into a water-cooled copper mould with a diameter of 30 mm and depth of 20 mm. During casting, the sample temperature was continuously measured and an average cooling rate of 60 °C/s was established. The annealing step was finished by quenching it in a water bath below 10 °C at an average cooling rate of 75 °C/s.

The investigated surface of the as-cast and annealed samples was subjected to grinding using up to 4000 grit abrasive papers. The surface topography was determined using a ZEISS LSM700 laser scanning confocal microscope (LSCM, Carl Zeiss AG, Oberkochen, Germany). The 405 nm light source was used, which in combination with a Epiplan-Apochromat 50×/0.95 objective enabled to reach step sizes of 250 nm on the X and Y axes as well as 200 nm on the Z axis. These surfaces were subjected to the corrosion in the salt chamber.

The investigated samples were coated with Lacomit Varnish to prevent the corrosion of the entire sample and limit the exposed area. The exposed surface was digitally scanned to double check the exposed area. These data, together with the surface topography data, made it possible to calculate the real surface area exposed to the corrosion on each sample.

The neutral salt spray corrosion test (NSST) was performed in a Co.Fo.Me.Gra 400E (CO.FO.ME.GRA. Srl, Milano, Italy) corrosion chamber according to the ISO 9227:2017 Standard [23]. The NSST samples were immediately exposed in the cabinet to a 5 wt.% NaCl solution. The air pressure of the atomized saline solution was maintained in the range of 95–105 kPa, and the temperature inside the cabinet was 35 ± 2 °C, pH level was 6.6–7.1, and the salt solution deposition rate 125–200 mL/h/m^2^. Custom holders were used to keep the prescribed sample orientation of 15° from the vertical position.

Exposure times for all types of samples were 250, 500, 750 and 1000 h. Three samples were prepared for all as-cast and annealed conditions for all exposure times. All in all, 144 individual samples were exposed at the same time. After the salt spray testing, the samples were dried at room temperature for 24 h at minimum before being further processed. After drying, loose corrosion products were removed and collected separately. It was of upmost importance to prevent any kind of a mechanical damage to the metallic sample surface. The bulk samples were cleaned by acetone and dried on air. The initial weight of the specimen was measured (*w*_0_) by using the Mettler Toledo XPR205 weighing balance (Mettler-Toledo International Inc., Columbus, OH, USA). According to the ASTM G31 Standard [24], the specimens were immersed in the chromate acid (CrO_3_) to ensure that the corrosion products were removed. Samples were cleaned in 60 s intervals. After each cleaning interval, the samples were repeatedly weighed. This process was considered finished when less than 5 mg of weight was lost after a cleaning cycle for all three repeats of a condition [23,24,25]. The final weight for each sample was recorded (*w_n_*). The recorded weight difference was normalized by the exposed area of each sample (*A_n_*) corrected by the sample topography coefficient (*k*). The topography coefficient is retrieved from LSCM software as the ratio between real surface area, incorporating surface topography, and the ideal surface. This value was 1.09 on average. These data enabled the calculation the average weight change (*w*′) for each condition in mg/mm^2^ according to equation:

(1) $$w^{\prime }=\frac{w_{0}-w_{n}}{A_{n}\;k}$$

The metallographic preparation on the longitudinal section (along the cylinder axis) of corroded as-cast and annealed samples consisted of standard grinding using abrasive papers, polishing on diamond pastes with various grain sizes down to 0.25 μm.

The microstructure evaluation was performed by the JEOL JSM 7600F scanning electron microscopy (SEM, Jeol Ltd., Tokyo, Japan) with a Schottky field emission electron source operating at 20kV and 90 µA. The samples were placed at a working distance of 15 mm and documented using a backscattered electron detector.

The quantitative analysis of IG corrosion depth was performed by ImageJ 1.53c software [26] along the longitudinal section for each condition. At least 150 individual values were recorded for each data point.

The weight measurements are displayed with +/− standard deviation error bars and the depth of IG corrosion measurements are given with +/− standard error.

The X-ray diffraction (XRD) analysis was carried out by the PANalytical Empyrean X-ray diffractometer (Malvern Panalytical Ltd., Malvern, UK) with configurations as detailed in Table 2. The measurements were performed on the samples after 1000 h of NSST with Ni filtered Cu-radiation. X-ray diffraction data were further analysed qualitatively using the PANalytical Xpert High Score program (HighScore Plus 3.0.5 version) with ICSD FIZ Karlsruhe database. These findings were confirmed and enhanced using the Rietveld refinement-based program, MAUD version 2.84 [27]. The program uses an asymmetric pseudo-Voight function to describe the experimental peaks. The instrument broadening was determined by measuring the NIST660c LaB_6_ (The National Institute of Standards and Technology, Gaithersburg, MD, USA) line position and line broadening standard and introduced to the Rietveld refinement program (MAUD version 2.84) via the Caglioti equation. An anisotropic size-strain model was applied to the majority of corrosion products, while the other phases were treated by isotropic models. A minor discrepancy between nominal and measured peak intensities was corrected using the spherical harmonic functions with fibre symmetry. The quality of the fit was in all analysed patterns achieved below 10% R_wp_.

## 3. Results

**Figure 1:**
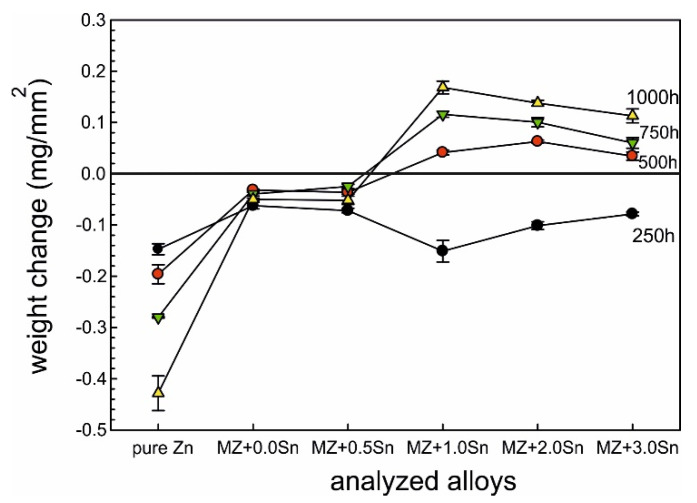
Weight change after corrosion measured on as-cast samples.

**Figure 2:**
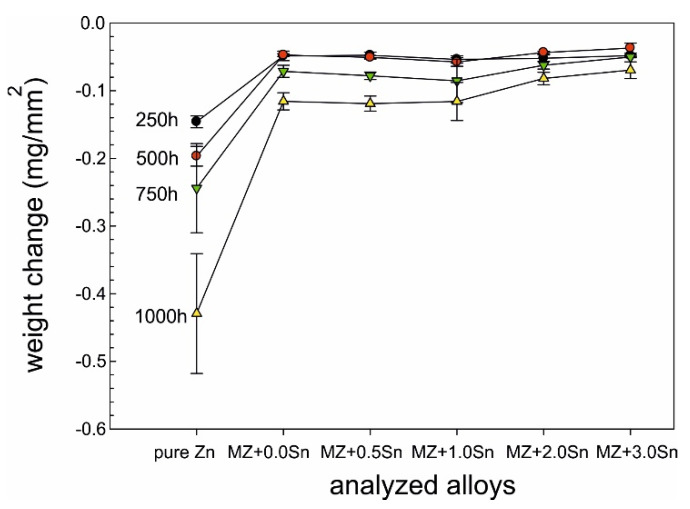
Weight change after corrosion measured on annealed samples.

As mentioned before, the weight changes for each sample were calculated according to Equation (1) and the obtained data are plotted in Figure 1 and Figure 2. Reference Zn samples showed a gradual weight loss for both as-cast and annealed conditions as expected. It can be observed that for several as-cast samples, a weight gain rather than a weight loss was recorded. The annealed samples showed the weight loss for all conditions as expected. MZ + 3.0Sn showed the best results at even 40% lower values compared to MZ + 0.0Sn.

**Figure 3:**
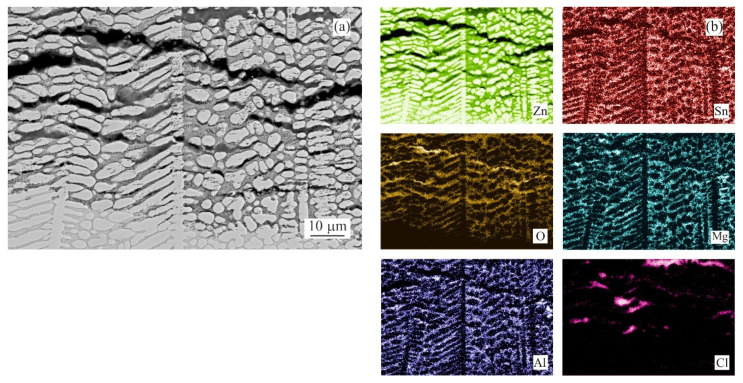
Anchoring effect of η(Zn) dendrites with corroded interdendritic spaces (MZ + 2.0Sn, as-cast): (**a**) overview BSEM image; (**b**) chemical element distribution maps of Zn, Sn, O, Mg, Al and Cl.

Since the weight gain instead of the weight loss was recorded for several as-cast conditions, it was decided to prepare longitudinal cuts of the samples and investigate potential reasons of this phenomena. The intergranular corrosion was present in most samples to a significant extent. Most phases present in the interdendritic spaces were corroded. Such corrosion products could not be cleaned by CrO_3_ acid solution [24]. These corrosion products, anchored among the still mainly intact η(Zn) dendrites, were increasing the total weight of the samples even after the cleaning process (Figure 3a). Their presence is visualised by chemical element distribution maps in Figure 3b.

**Figure 4:**
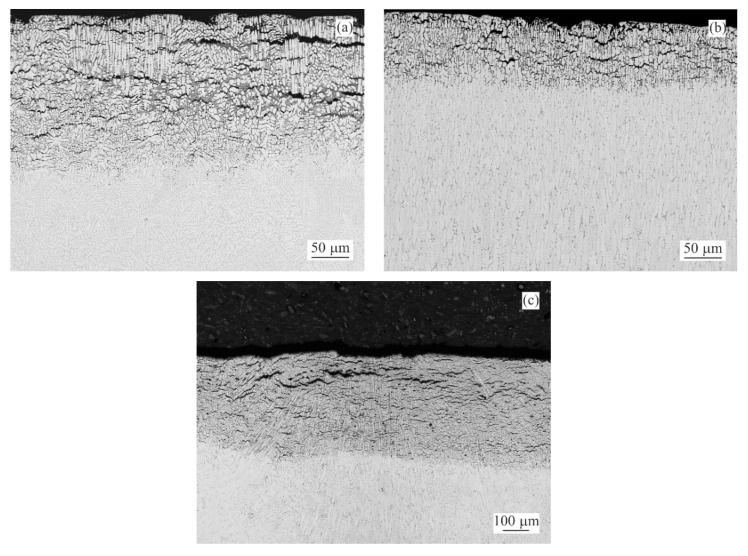
BSEM images indicating the extend of IG corrosion observed for the as-cast samples after 1000 h of NSST: (**a**) MZ + 0.0Sn (**b**) MZ + 0.5Sn (**c**) MZ + 3.0Sn.

**Figure 5:**
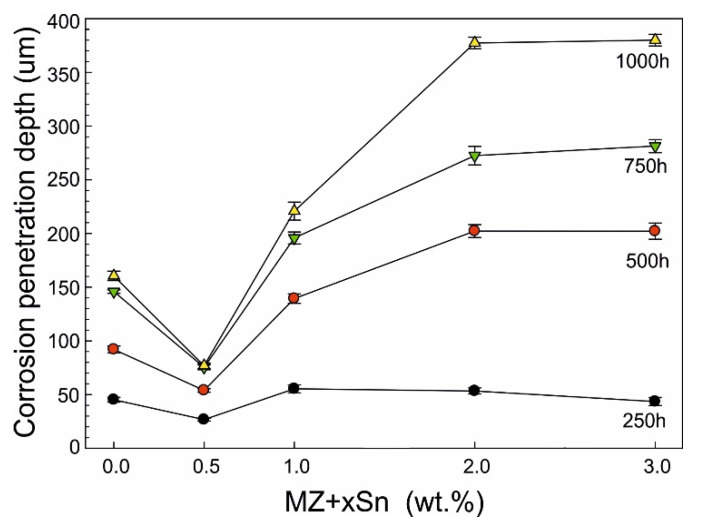
Depth of IG corrosion—as-cast samples.

**Figure 6:**
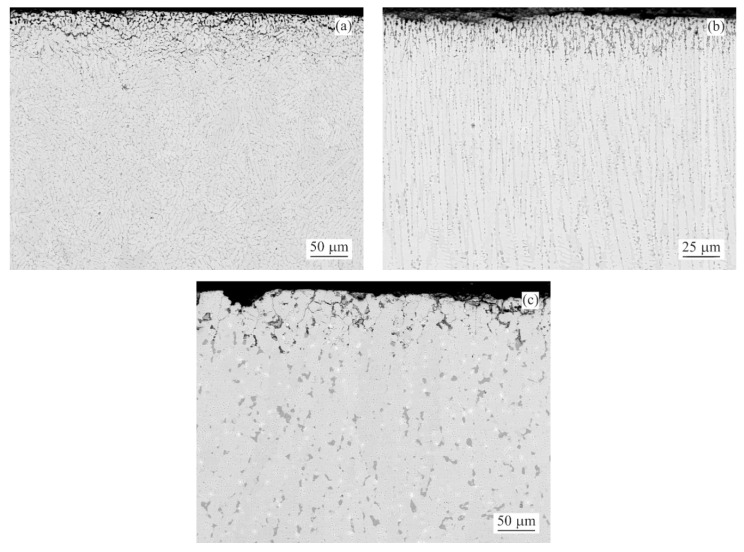
BSEM images indicating the extent of IG corrosion observed for the annealed samples: (**a**) MZ + 0.0Sn (**b**) MZ + 0.5Sn (**c**) MZ + 3.0Sn.

**Figure 7:**
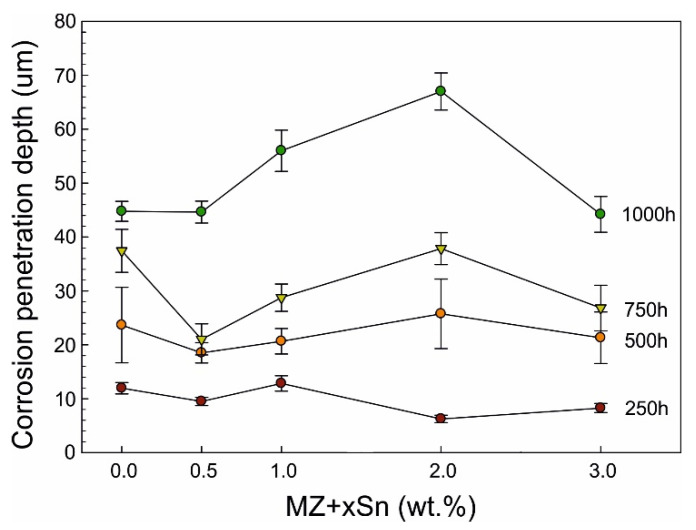
Depth of IG corrosion—annealed samples.

Backscattered-electron scanning electron microscopy (BSEM) images of the longitudinal sections for representative as-cast samples with 0.0, 0.5 and 3.0 wt.% of Sn after 1000 h of NSST are given in Figure 4. Corresponding quantitative analysis results of the intergranular corrosion penetration depth are summarized in Figure 5. The same is available for the annealed samples in Figure 6 and Figure 7.

**Figure 8:**
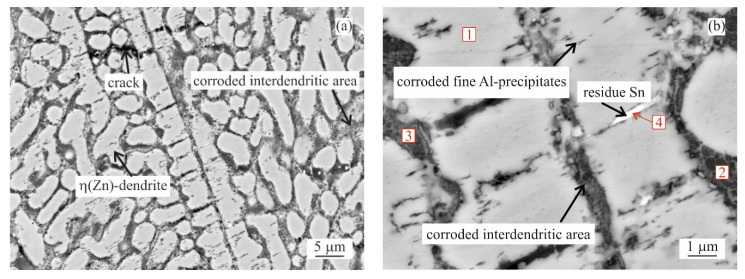
MZ + 2.0Sn as-cast microstructure after 1000 h in NSST affected by IG corrosion: (**a**) overview, (**b**) detail.

SEM investigation of as-cast MZ + 0.0Sn samples after 250 h of NSST revealed a significant portion of the microstructure being affected by the intergranular corrosion reaching as deep as ~50 μm (Figure 5). This effect is even more pronounced on the as-cast samples with 1–3 wt.% of Sn. IG corrosion can be formed as deep as ~150 μm for the as-cast MZ + 3.0Sn samples (Figure 5). This effect is further emphasised during longer exposures in the salt spray chamber. The IG corrosion can reach depths of over 370 μm on average for the as-cast MZ + 2.0Sn and MZ + 3.0Sn samples exposed for 1000 h (Figure 4c and Figure 5). Complex ZnAlMg interdendritic areas were affected preferentially by the IG corrosion (Figure 8).

The depth of IG corrosion is significantly lower for the annealed samples with maximums reaching only about 70 µm even after 1000 h of NSST. For 250 and 500 h, all alloys behaved rather similar with IG corrosion depths of 10 and 22 µm, respectively. MZ + 1.0Sn and MZ + 2.0Sn seem to be more susceptible to the IG corrosion when comparing the samples after the full 1000 h test. On the contrary, annealed MZ + 3.0Sn samples showed values comparable even to MZ + 0.0Sn, or MZ + 0.5Sn reaching a maximum depth of about 45 µm.

**Figure 9:**
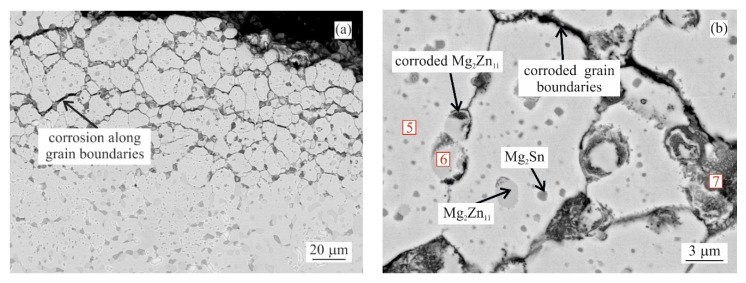
MZ + 2.0Sn annealed microstructure after 1000 h in NSST affected by IG corrosion: (**a**) overview, (**b**) detail.

The examples of areas affected by the intergranular corrosion are given in Figure 8 and Figure 9 for the as-cast and annealed samples, respectively. The EDS chemical analysis of the microstructure in Table 3 confirms the intergranular corrosion attack. Figure 8a shows the η(Zn) dendritic microstructure affected by the corrosion along the interdendritic areas. η(Zn) primary dendrites also showed the signs of corrosion in the form of fine cracks. These can be attributed to the presence of fine, sub-micron Al-rich particles observed within the η(Zn) primary dendrites. In a more detailed image (Figure 8b) it can be seen that α(Al) and Mg_x_Zn_y_ particles were corroded. Mg_2_Sn particles were subject to the process of dealloying [28,29,30,31], leaving thus pure metallic Sn particles behind.

As reported in the first part of this research [22], the basic dendritic character of the microstructure was still rather well maintained for the MZ + 0.0Sn and MZ + 0.5Sn alloys even after annealing. Hence, the IG corrosion is observed to propagate preferably along the interdendritic areas. For the annealed samples with 1 and more wt.% of Sn the microstructure is more spheroidized. The individual intermetallic phases were coalesced into coarse, discrete particles, while η(Zn) dendrites were reshaped and new grains are formed within the microstructure. The boundaries of these grains contained a significant portion of intermetallic phase particles. As such, they were more susceptible to the IG corrosion. The propagation of the IG corrosion is documented in Figure 9a and the grain boundaries decorated by intermetallic particles are shown in closer detail in Figure 9b.

**Figure 10:**
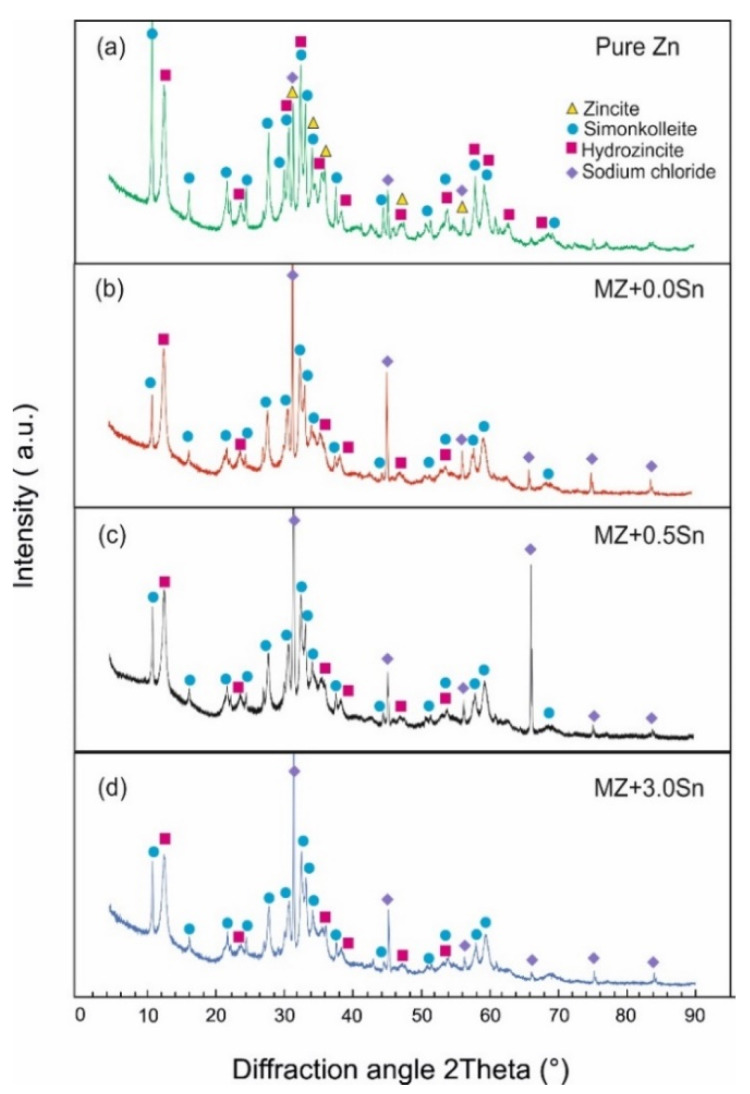
XRD patterns of corrosion product powders retrieved from the as-cast samples after 1000 h of NSST: (**a**) pure Zn (**b**) MZ + 0.0Sn (**c**) MZ + 0.5Sn (**d**) MZ + 3.0Sn.

**Figure 11:**
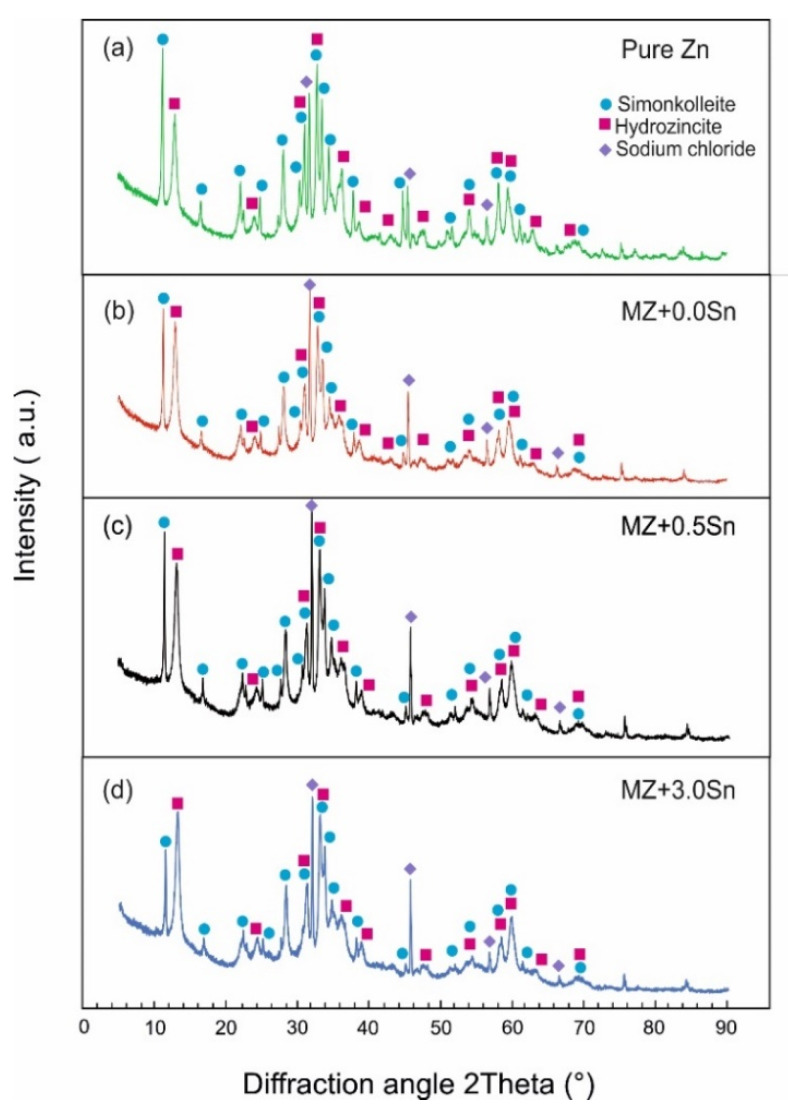
XRD patterns of corrosion product powders retrieved from the annealed samples after 1000 h of NSST. (**a**) pure Zn (**b**) MZ + 0.0Sn (**c**) MZ + 0.5Sn (**d**) MZ + 3.0Sn.

The XRD analysis was performed on all samples after NSST. As described, loose corrosion products were gathered and investigated. The XRD was used to determine the phase composition of the corrosion products formed on the samples during NSST. The XRD patterns for all corrosion products retrieved from the as-cast and annealed samples are summarized in Figure 10 and Figure 11, respectively. The presence of the identified phases was also confirmed using the Rietveld method (Table 4). Despite the differences between the microstructure of the as-cast and annealed samples, their corrosion products showed an identical qualitative phase composition. The semi-quantitative results from these calculations indicate that the majority of the corrosion products were formed by a hydrozincite for all samples. About 20 vol.% of simonkolleite was measured for all pure Zn samples (Figure 10a and Figure 11a). The corrosion products of MZ-based samples contained only about 10 vol.% of simonkolleite on average (Figure 10b–d and Figure 11b–d).

ZnO was identified solely in the corrosion products of the pure as-cast Zn sample (Figure 10a), representing only about 2 vol.%.

NaCl was identified in randomly varying amounts in the corrosion products as a remainder of the corrosion environment.

**Figure 12:**
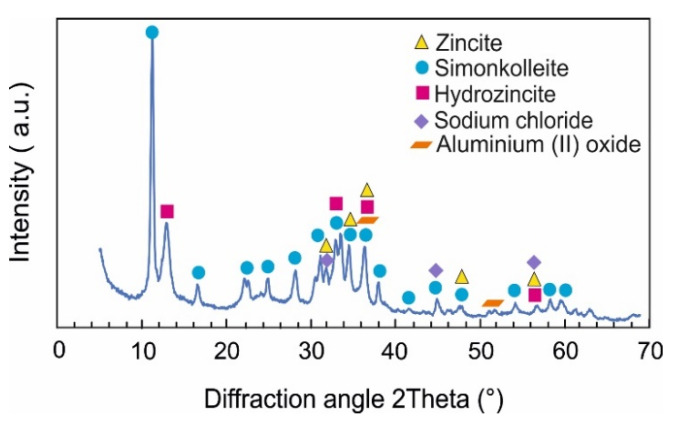
XRD pattern on MZ + 3.0Sn annealed sample surface after 1000 h NSST, measured in grazing incident geometry.

Next to hydrozincite and simonkolleite, the sources indicated that other phases might also be formed [17,18,19,20,32,33,34,35,36,37,38,39]. Therefore, the corroded surfaces of the bulk metallic samples were investigated after the loose corrosion products were removed. The measurement in grazing incident diffraction mode with 0.5° incident angle was chosen to limit the signal from the substrate (mainly Zn) as much as possible. Additionally, to previously identified phases, zincite (ZnO) and aluminium (II) oxide (AlO) were identified as present directly attached to the sample surface. An example of such a pattern is given in Figure 12 for the MZ + 3.0Sn annealed sample surface after 1000 h of NSST. However, only about 2 and 5 vol.% of ZnO and AlO, respectively, were identified using the Rietveld method.

## 4. Discussion

### 4.1. SEM vs. Mass Balance after NSST

**Figure 13:**
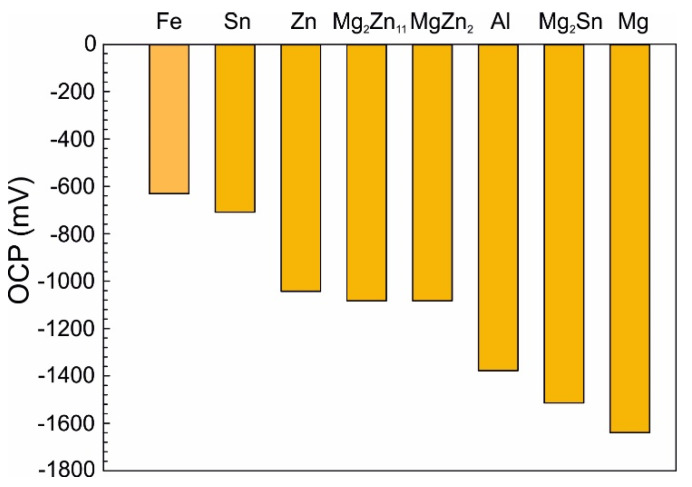
Overview of OCP values for phases present in the investigated system [34,40,41,42,43,44].

Both weight loss and weight gain were observed for a significant portion of the as-cast samples due to several related properties of the ZnAlMg alloy system. During directional cooling of these alloys, the η(Zn) phase forms the primary dendrites, while the interdendritic spaces are formed by a fine mixture of various phases including α(Al) solid solution, MgZn_2_, Mg_2_Zn_11_, and Mg_2_Sn intermetallic phases. There is an inherent difference in the open circuit potential (OCP) of these phases mainly compared to the η(Zn) phase (Figure 13). Consequently, the interdedritic phases seem to offer the galvanic protection to the η(Zn) phase dendrites. Due to this phenomenon, the interdendritic spaces corrode prior to the η(Zn) phase. The still intact η(Zn) phase dendrites act as anchors holding these corrosion products in place. These corrosion products cannot be removed by the environment during the NSST, nor by chemical cleaning done in the preparation for the sample weighing after the test. Naturally, the total weight of such corrosion products is greater as the weight of the original metallic phases. This phenomenon will cause weight gain for several samples even after the corrosion products were removed as much as possible before weighing. This increase in weight is also followed by an increase in volume. Following the BSEM images, it is clear that the η(Zn) phase dendrites are cracking as seen in Figure 8. This could be attributed to volume expansion-induced cracking (Figure 3a and Figure 8a).

### 4.2. Corrosion of Individual Phases

Based on the SEM investigation, it can be concluded that individual phases are corroding in the following order: α(Al) → Mg_x_Zn_y_ → Mg_2_Sn (if present) → η(Zn).

**Figure 14:**
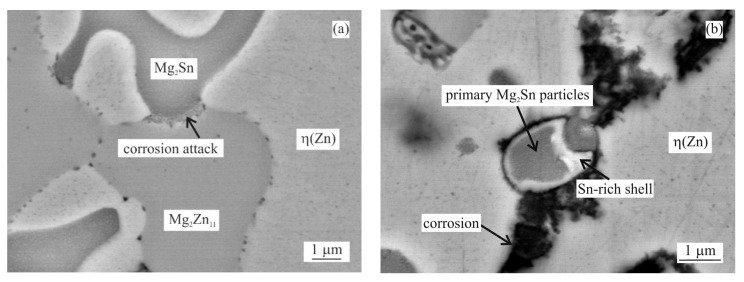
Behaviour of Mg_2_Sn intermetallic phase during corrosion (MZ + 2.0Sn, annealed): (**a**) Influence of Mg_2_Sn on initial stage of corrosion of Mg_2_Zn_11_ intermetallic particle; (**b**) dealloying of Mg_2_Sn particle.

**Figure 15:**
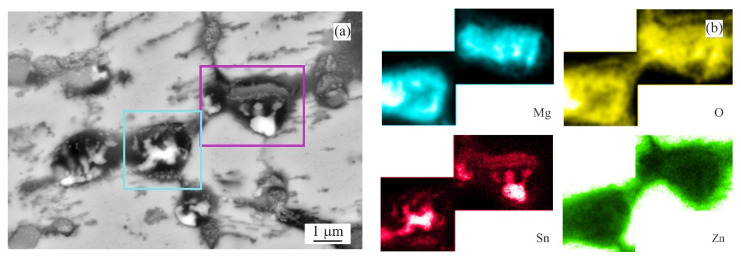
Details of Mg_2_Sn particles affected by dealloying (MZ + 3.0Sn, annealed): (**a**) overview BSEM image; (**b**) chemical element distribution maps of Mg, O, Sn and Zn.

For α(Al), Mg_x_Zn_y_ and η(Zn) this order corresponds with their respective corrosion potentials reported in the literature as seen in Figure 13. On the contrary, Mg_2_Sn behaves as a more noble phase compared to α(Al) and Mg_x_Zn_y_ phases despite having a lower OCP compared to these phases. A clear example is given in Figure 14a, where already in an early stage of the corrosion attack, an Mg_2_Zn_11_ particle is affected by the corrosion when in contact with an Mg_2_Sn particle. Such a phenomenon can be caused by several factors, such as for example a local change in pH, or local change in chemical composition of these Mg_2_Sn particles. The second phenomenon was regularly observed in all Sn-containing alloys. During the corrosion, the local dealloying of Mg_2_Sn particles occurs. Mg_2_Sn particles are separated into Mg and Sn atoms. Mg is most probably immediately forming new corrosion products, while Sn resides in the form of metallic particles. These can be observed on most BSEM images of the areas affected by the IG corrosion. As shown in Figure 14b, even the formation of an Sn-rich shell can be observed on larger particles found in the annealed samples. The Mg content of former Mg_2_Sn particles is being gradually reduced, hence, the remaining metallic particle will have locally a higher potential compared to neighbouring microstructure components. As a final stage, pure Sn particles are formed in the place of Mg_2_Sn particles. This process is even described by several authors [28,29,30,31] as a potential energy storage system for batteries. The final stage of this process is documented in Figure 15a. The corresponding EDS maps in Figure 15b confirm the presence of Sn-based metallic particles. Mg and O maps are overlapping, indicating that Mg is forming corrosion products.

### 4.3. Phase Composition of Corrosion Products

Hydrozincite and simonkolleite are the most common corrosion products reported by several authors for similar alloy systems [18,19,20,32,33,36,37,38]. This is in good agreement with the current results.

The semi-quantitative analysis showed that a slightly higher portion of simonkolleite was found in the corrosion products of both as-cast and annealed pure Zn samples compared to MZ + xSn alloys. This is in line with the observation of Prosek et al. [32], where simonkolleite was more likely to be identified for pure Zn coatings. As the XRD analysis could not give the data on the chemical composition of these phases, the presence of Mg and Al in the corrosion products of MZ-based alloys was measured by the SEM EDS analysis with up to 2 wt.% of Mg and up to 1 wt.% of Al. From the two main corrosion products, the hydrozincite can accommodate Mg as a metallic ion in its structure [45,46]. This would support our observation, where the increased amount of the hydrozincite was identified on the MZ + xSn samples compared to pure Zn.

Additionally, layered double hydroxide (LDH) phases were identified, where LDH can represent a group of similar phases [18,19,20,32,36,39,47]. Azevedo et al. [20] identified LDH within the corrosion products formed on a Zn3.7Al3.0Mg alloy coating after 100 h of NSST (5% NaCl). Applying the Rietveld method refinement to their XRD pattern revealed that about 2–3 vol.% of the corrosion products were formed by LDH (ICSD FIZ Karlsruhe database 98-015-5051). Similarly, a low amount of LDH was indicated by the semi-quantitative results of Prosek et al. [32] within the corrosion products of Zn1.5Al1.5Mg alloy coatings exposed to model atmospheric conditions. However, in the studied system, LDH was not confirmed in any of our measurements, not even during GI XRD measurements performed directly on the corroded surface with the loose corrosion products removed. When comparing our experiments to the literature, there are two probable causes: we had rather low Al and Mg content compared to NSST done by Azevedo et al. [20]. Prosek et al. [32] used the same coating, however, in a very different corrosion environment. We might have a specific combination of parameters, which are not favourable for the creation of LDH.

## 5. Conclusions

Based on the experimental results discussed in part I and part II of this research, the following conclusions can be drawn:

- Weight change cannot be correlated with alloy composition nor NSST exposure time due to presence of IG corrosion.
- Increasing the exposure time in NSST from 250 h to 1000 h increases the intergranular corrosion penetration depth, regardless of the chemical composition and heat treatment.
- As-cast samples were more susceptible to the IG corrosion as interdendritic areas are forming a connected network of less noble phases. These include α(Al), Mg_x_Zn_y_ and Mg_2_Sn, while dendrites are formed mainly by η(Zn).
- Adding 0.5 wt.% Sn has almost no effect on the weight change of the as-cast samples after NSST compared to MZ + 0.0Sn, while being significantly less susceptible to the IG corrosion. As a result, the as-cast MZ + 0.5Sn samples show the most favourable corrosion behaviour.
- Adding 1 to 3 wt.% of Sn yields in the weight gain instead of the weight loss as well as a significant increase in the IG corrosion depth.
- Annealed alloys are less susceptible to the IG corrosion as intermetallic phases are coalesced, spheroidised, and more uniformly distributed within the η(Zn) matrix or at newly formed grain boundaries of η(Zn).
- Changing the alloy composition of the annealed samples has only a slight effect on the weight change. Nevertheless, the samples with 3 wt.% of Sn showed the most favourable results. Meanwhile, the IG corrosion depth is comparable to MZ + 0.0Sn, resulting in overall best performance of the annealed MZ + 3.0Sn samples.
- Hydrozincite and simonkolleite were identified as the main corrosion products on all samples. A small portion of ZnO was identified only on pure Zn samples. GI XRD measurements indicated a small amount of AlO formed on most MZ-based samples.
- Current results show that even the high temperature exposure of up to 310 °C does not negatively affect the corrosion performance of these alloys. It could be noted that such exposure even provides a beneficial effect and enhances the corrosion resistance of the coating by suppressing the IG corrosion.

## Figures and Tables

**Table 1:** Chemical composition of the studied alloys (wt.%).

Alloy	Al	Mg	Sn	Zn
MZ + 0.0Sn	1.56 ± 0.07	1.40 ± 0.01	0.07 ± 0.02	bal.
MZ + 0.5Sn	1.64 ± 0.02	1.41 ± 0.01	0.52 ± 0.01	bal.
MZ + 1.0Sn	1.62 ± 0.03	1.45 ± 0.02	1.06 ± 0.02	bal.
MZ + 2.0Sn	1.57 ± 0.01	1.44 ± 0.01	1.95 ± 0.01	bal.
MZ + 3.0Sn	1.57 ± 0.12	1.43 ± 0.05	2.69 ± 0.06	bal.

**Table 2:** List of XRD measurements settings.

Sample	XRD Device Geometry	Angle Range	Incident Beam	Diffracted Beam	Detector
Powder of loose corrosion products scraped from the surface of the bulk samples	Theta-2Theta, Bragg-Brentano geometry	5°–90° 2Theta	Divergence slit: 1/4° Soller slit: 0.04 rad Anti-scatter slit: 1/2°	Anti-scatter slit: 1/2° Soller slit: 0.04 rad	PIXcel3D detector in 1D scanning mode
Corroded surface of bulk samples after loosely attached corrosion products were removed	Grazing incident (GI) with 0.5° incident angle	5°–80° 2Theta	Parallel beam optics with: Divergence slit: 1/16° Soller slit: 0.04 rad	Parallel plate collimator: 0.27° Soller slit: 0.04 rad	Scintillation detector

**Table 3:** EDS chemical composition of selected sites (at.%).

Site No. (as Labeled in Figure 8 and Figure 9)							
Chemical Element (at.%)	1	2	3	4	5	6	7
Zn	97.31	55.32	29.66	46.12	96.08	77.88	21.29
Al	2.69	2.69	5.31	2.83	2.53	2.54	30.42
Mg	0.00	5.11	13.23	2.88	0.00	17.38	1.00
Sn	0.00	0.38	0.25	14.05	0.00	0.00	0.98
O	0.00	33.15	59.95	33.97	1.39	2.20	46.13
Cl	0.00	3.34	0.60	0.14	0.00	0.00	0.19
Phase/Region	η(Zn)	Corrosion product	Corrosion product	Residue Sn from Mg_2_Sn particle	η(Zn)	Mg_2_Zn_11_	Corrosion product

**Table 4:** Phases identified during XRD analysis.

Phase Name	Phase Chemical Formula	Reference Code—ICSD FIZ Karlsruhe Database	Crystallography Open Database COD ID	Crystal System	Space Group	Space Group Number
Hydrozincite	Zn_5_(OH)_6_(CO_3_)_2_	01-072-1100	9007481	Monoclinic	C2/m	12
Simonkolleite	Zn_5_(OH)_8_Cl_2_·H_2_O	98-003-4904	9004683	Hexagonal	$R\bar{3}m$	166
Zincite	ZnO	98-015-4487	9004178	Hexagonal	$P6_{3}/\mathrm{mc}$	186
Aluminium (II) Oxide	AlO	98-002-8920	-	Cubic	$\mathrm{Fm}\bar{3}m$	225
Sodium Chloride	NaCl	01-075-0306	1000041	Cubic	$\mathrm{Fm}\bar{3}m$	225

## Data Availability

Data sharing is not applicable.

## References

[1] Vida T.A., Brito C., Lima T.S., Spinelli J.E., Cheung N. (2009). Near-eutectic Zn-Mg alloys: Interrelations of solidification thermal parameters, microstructure length scale and tensile/corrosion properties. Curr. Appl Phys..

[2] Tokuda S., Muto I., Sugawara Y., Takahashi M., Matsumoto M., Hara N. (2017). Micro-electrochemical investigation on the role of Mg in sacrificial corrosion protection of 55mass%Al-Zn-Mg coated steel. Corros. Sci..

[3] Krystýnová M., Doležal P., Fintová S., Zapletal J., Marada T., Wasserbauer J. (2018). Characterization of Brittle Phase in Magnesium Based Materials Prepared by Powder Metallurgy. Key Eng. Mater.

[4] Vida T.A., Soares T., Septimio R.S., Brito C.C., Cheung N., Garcia A. (2019). Effects of Macrosegregation and Microstructure on the Corrosion Resistance and Hardness of a Directionally Solidified Zn-5.0wt.%Mg Alloy. Mater. Res..

[5] Pinc J., Čapek J., Kubásek J., Veřtát P., Hosová K. (2019). Microstructure and mechanical properties of the potentially biodegradable ternary system Zn-Mg0.8-Ca0.2. Procedia Struct. Integr..

[6] De Bruycker E., Zermout Z., De Cooman B.C. (2007). Zn-Al-Mg Coatings—Thermodynamic Analysis and Microstructure Related Properties. Mater. Sci. Forum.

[7] De Bruycker E., De Cooman B.C., De Meyer M. (2005). Experimental study and microstructure simulation of Zn-Al-Mg coatings. Rev. Metall-CIT.

[8] Akdeniz V.M., Wood J.V. (1996). Microstructures and phase selection in rapidly solidified Zn-Mg alloys. J. Mater. Sci..

[9] Liu H.Y., Jones H. (1992). Solidification Microstructure Selection and Characteristics in the Zinc-Based Zn-Mg System. Acta Metall. Mater..

[10] Prosek T., Nazarov A., Goodwin F., Šerák J., Thierry D. (2016). Improving corrosion stability of Zn-Al-Mg by alloying for protection of car bodies. Surf. Coat. Technol..

[11] Farahany S., Tat L.H., Hamzah E., Bakhsheshi-Rad H.R., Cho M.H. (2017). Microstructure development, phase reaction characteristics and properties of quaternary Zn-0.5Al-0.5Mg-xBi hot dipped coating alloy under slow and fast cooling rates. Surf. Coat. Technol..

[12] Gondek J., Babinec M., Kusý M. (2015). The corrosion performance of Zn-Al-Mg based alloys with tin addition in neutral salt spray environment. J. Achiev. Mater. Manuf. Eng..

[13] Vargel C. (2004). Corrosion of Aluminium.

[14] Volovitch P., Allely C., Ogle K. (2009). Understanding corrosion via corrosion product characterization: I Case study of the role of Mg alloying in Zn–Mg coating on steel. Corros. Sci..

[15] Odnevall W., Leygraf C. (2017). A Critical Review on Corrosion and Runoff from Zinc and Zinc-Based Alloys in Atmospheric Environments. Corros. J. Sci. Eng..

[16] De la Fuente D., Castaño J.G., Morcillo M. (2007). Long-term atmospheric corrosion of zinc. Corros. Sci..

[17] Volovitch P., Vu T.N., Allély C., Abdel Aal A., Ogle K. (2011). Understanding corrosion via corrosion product characterization: II Role of alloying elements in improving the corrosion resistance of Zn–Al–Mg coatings on steel. Corros. Sci..

[18] Thierry D., Persson D., Luckeneder G., Stellnberger K.-H. (2019). Atmospheric corrosion of ZnAlMg coated steel during long term atmospheric weathering at different worldwide exposure sites. Corros. Sci..

[19] LeBozec N., Thierry D., Persson D., Riener C.K., Luckeneder G. (2019). Influence of microstructure of zinc-aluminium-magnesium alloy coated steel on the corrosion behavior in outdoor marine atmosphere. Surf. Coat. Technol..

[20] Azevedo M.S., Allély C., Ogle K., Volovitch P. (2015). Corrosion mechanisms of Zn(Mg, Al) coated steel in accelerated tests and natural exposure 1. The role of electrolyte composition in the nature of corrosion products and relative corrosion rate. Corros. Sci..

[21] Ghosh P., Mezbahul-Islam M., Medraj M. (2012). Critical assessment and thermodynamic modeling of Mg-Zn, Mg-Sn, Sn-Zn and Mg-Sn-Zn systems. Calphad.

[22] Gogola P., Gabalcová Z., Kusý M., Suchánek H. (2021). The effect of Sn addition on Zn-Al-Mg alloy; Part I: Microstructure and phase composition. Materials.

[23] (2017). Corrosion Tests in Artificial Atmospheres-Salt Spray Tests.

[24] (2021). Standard Guide for Laboratory Immersion Corrosion Testing of Metals.

[25] Jokar M., Aliofkhazraei M. (2017). Comprehensive Materials Finishing.

[26] Schneider C.A., Rasband W.S., Eliceiri K.W. (2012). NIH Image to ImageJ: 25 Years of image analysis. Nat. Methods.

[27] Lutterotti L., Matthies S., Wenk H.R. MAUD (Material Analysis Using Diffraction): A user friendly Java program for rietveld texture analysis and more. Proceedings of the 12th International Conference on Textures of Materials (ICOTOM-12), McGill University Montreal.

[28] Nguyen G.T.H., Nguyen D.-T., Song S.-W. (2018). Unveiling the Roles of Formation Process in Improving Cycling Performance of Magnesium Stannide Composite Anode for Magnesium-Ion Batteries. Adv. Mater. Interfaces.

[29] Nguyen D.-T., Song S.-W. (2017). Magnesium stannide as a high-capacity anode for magnesium-ion batteries. J. Power Sources.

[30] Singh N., Arthur T.S., Ling C., Matsui M., Mizuno F. (2013). A high energy-density tin anode for rechargeable magnesium-ion batteries. Chem. Commun..

[31] Yaghoobnejad Asl H., Fu J., Kumar H., Welborn S.S., Shenoy V.B. (2018). In Situ Dealloying of Bulk Mg_2_Sn in Mg-Ion Half Cell as an Effective Route to Nanostructured Sn for High Performance Mg-Ion Battery Anodes. Chem. Mater..

[32] Prosek T., Persson D., Stoulil J., Thierry D. (2014). Composition of corrosion products formed on Zn–Mg, Zn–Al and Zn–Al–Mg coatings in model atmospheric conditions. Corros. Sci..

[33] Zhang X.G. (1996). Corrosion and Electrochemistry of Zinc.

[34] Buyn J.M., Yu J.M., Kim D.K., Kim T.-Y., Jung W.-S., Kim Y.D. (2012). Corrosion Behavior of Mg_2_Zn_11_ and MgZn_2_ Single Phases. Korean J. Met. Mater..

[35] McMahon M.E., Burns T.J., Scully J.R. (2019). Development of new criteria for evaluating the effectiveness of Zn-rich primers in protecting Al-Mg alloys. Prog. Org. Coat..

[36] Persson D., Thierry D., LeBozec N., Prosek T. (2013). In situ infrared reflection spectroscopy studies of the initial atmospheric corrosion of Zn–Al–Mg coated steel. Corros. Sci..

[37] Li B., Dong A., Zhu G., Chu S., Qian H., Hu C., Sun B., Wang J. (2012). Investigation of the corrosion behaviors of continuously hot-dip galvanizing Zn–Mg coating. Surf. Coat. Technol..

[38] Zhu Z., Li A., Xu B. (2011). Study on corrosion mechanism of arc sprayed Zn-Al-Mg coatings by XRD and EIS. Adv. Mater. Res..

[39] Ennadi A., Legrouri A., De Roy A., Besse J.P. (2000). X-ray Diffraction Pattern Simulation for Thermally Treated [Zn-Al-Cl] Layered Double Hydroxide. J. Solid State Chem..

[40] Singh I.B., Singh M., Das S. (2015). A comparative corrosion behavior of Mg, AZ31 and AZ91 alloys in 3.5 NaCl solution. J. Magnesium Alloys.

[41] Špoták M., Drienovský M., Rízeková-Trnková L., Palcut M. Corrosion of Candidate Lead-Free Solder Alloys in Saline Solution. Proceedings of the 24th International Conference on Metallurgy and Materials METAL 2015.

[42] Hu C.-C., Wang C.-K. (2006). Effects of composition and reflowing on the corrosion behavior of Sn–Zn deposits in brine media. Electrochim. Acta.

[43] Calabrese L., Bonaccorsi L., Capri A., Proverbio E. (2014). Electrochemical behavior of hydrophobic silane-zeolite coatings for corrosion protection of aluminum substrate. J. Coat. Technol. Res..

[44] Gogola P., Gabalcová Z., Palcut P. Experimental determination of the corrosion potential for the intermetallic Mg_2_Sn phase.

[45] Mindat.org Hydrozincite. https://www.mindat.org/min-1993.html.

[46] Mindat.org Simonkolleite. https://www.mindat.org/min-3668.html.

[47] Zhitova E.S., Krivovichev S.V., Pekov I., Greenwell H.C. (2019). Crystal chemistry of natural layered double hydroxides. 5. Single-crystal structure refinement of hydrotalcite, [Mg_6_Al_2_(OH)_16_](CO_3_)(H_2_O)_4_. Mineral. Mag..

